# Introducing the clinical trials monitor: a new section of the journal for immunotherapy of cancer

**DOI:** 10.1186/s40425-015-0092-y

**Published:** 2015-10-20

**Authors:** Leisha A. Emens, Pedro Romero

**Affiliations:** Department of Oncology, Johns Hopkins University School of Medicine, The Kimmel Cancer Center at Johns Hopkins, Baltimore, MD USA; Translational Tumor Immunology Group, Ludwig Cancer Research Center, University of Lausanne, Lausanne, Switzerland

## Background

Since its inception, the Journal for Immunotherapy of Cancer (JITC) has focused on all aspects of tumor immunology and cancer immunotherapy from bench to bedside. Clinical advances in immuno-oncology are accelerating, with an increasing number of approved drugs that specifically target the immune system to treat cancer. The success of these agents in the clinic has given rise to a rapidly expanding portfolio of trials testing cancer immunotherapies in patients with a variety of cancer types. Given the explosion of interest and clinical activity in cancer immunotherapy, we are pleased to launch a new section of JITC, the Clinical Trials Monitor, edited by Leisha A. Emens, MD PhD, of the Johns Hopkins University.

## Introducing the section

The Clinical Trials Monitor section aims to publish and provide perspectives on late stage clinical trial results, highlight immuno-oncology trials in progress, and summarize important new regulatory approvals in cancer immunotherapy. The section also aims to address trial design and analysis considerations that may be unique to cancer immunotherapy, and to provide guidance for implementing and managing the use of cancer immunotherapies in the clinic. The section will disseminate clinically oriented information and perspectives in the following formats:

### Clinical trial reports

This feature will include Phase 2 or 3 clinical trials with primary clinical endpoints, studies that define biomarkers of therapeutic response, primary resistance to immunotherapy, and immune escape, and trials that define the patient experience (quality of life) with cancer immunotherapies.

### Into the clinic

This feature will review newly approved immunotherapies, highlighting the drug development process for the agent and indication, including any important biomarkers. It will also provide guidance about how the newly approved drug fits into the standard of care as it enters the clinic. These pieces will be written by leading immunotherapy experts in disease-specific areas, and should help inform the development of clinical practice guidelines by SITC, NCCN, and ASCO.

### Regulatory approval summary

This feature will include approval summaries from regulatory agencies around the world as new immunotherapies are approved for standard clinical use.

## Cutting edge: Immunotherapy clinical trials in progress

This feature will highlight clinical trials grouped by modality, pathway, or disease subtype. These summaries will include an introduction followed by clinical trials in progress within the domain of focus. They will span early first-in-man single agent (novel immune checkpoint targets or innovative vaccine strategies) or important combination strategies (IDO inhibition with vaccines or immune checkpoint blockade), and later stage trials testing novel combinations with more established immunotherapies (radiation with immune checkpoint blockade).

### Glimpse of the future: emerging immunotherapies

These pieces will succinctly highlight mechanistic principles underlying emerging immunotherapeutic strategies and technologies entering the clinic, providing a sound scientific foundation for trial design and evaluating the preclinical and clinical literature.

### Beyond the numbers

This educational feature puts cancer immunotherapy into broad context. Subject matter may include a discussion of statistical considerations and perspectives essential for understanding results of pivotal clinical trials, clinical trial design and data analysis issues particularly relevant to cancer immunotherapy, and unresolved issues related to reduction to practice (toxicity management or issues related to access to novel immunotherapies (adoptive T cell therapy/CAR T cells).

## Conclusions

The Clinical Trials Monitor section will serve as a valuable resource for researchers and clinicians. It will report important new trial results, provide timely updates on new drug approvals and important ongoing clinical studies, and deliver key perspectives on clinical issues and advances in the field relevant to the everyday use of cancer immunotherapy in the clinic. A talented group of Editorial Board members from around the world with broad expertise in immuno-oncology will oversee the section. We expect to adapt the section to meet the changing needs of the field and the JITC readership, and we invite your feedback. These are exciting times for cancer immunotherapy, and we at JITC share your interest and enthusiasm for using cancer immunotherapy to transform cancer medicine and change the lives of cancer patients.

